# Outcomes of Secondary Ovulation Induction Following Failed Oocyte Pick-Up in In Vitro Fertilization (IVF) Cycles: A Retrospective Observational Study

**DOI:** 10.7759/cureus.87551

**Published:** 2025-07-08

**Authors:** Feras Sendy, Robert Hemmings, Isaac-Jacques Kadoch, Simon Phillips

**Affiliations:** 1 Obstetrics and Gynecology, King Fahad Medical City, Riyadh, SAU; 2 Obstetrics and Gynecology, University of Montreal Health Centre (CHUM), Montreal, CAN; 3 Reproductive Endocrinology and Infertility, Clinique Ovo, Montreal, CAN

**Keywords:** blastocyst, failed oocyte pick-up, fertilization, oocyte retrieval, pregnancy

## Abstract

Introduction: Failed oocyte pick-up (FOPU) is the absence of oocyte retrieval from mature follicles after ovulation induction in in vitro fertilization (IVF) cycles. The incidence of FOPU is rare in IVF cycles.

Methods: This retrospective cohort single-center study compared a 36-hour time interval between a second trigger and ovum pick-up (OPU) in FOPU and a 24-hour time interval between a second trigger and OPU in FOPU. In total, 62 couples with FOPU between July 2011 and April 2024 were included in the study. Of the 62 patients included, 49 underwent a 36-hour time interval between trigger and OPU (group 1), and 13 couples underwent a 24-hour time interval between trigger and OPU (group 2). The primary outcome measured was oocyte retrieval, and the secondary outcomes were fertilization, blastocyst, and pregnancy rates.

Results: We compared 49 patients from group 1 and 13 from group 2. After a 36-hour time interval, oocyte retrieval in group 1 was significantly higher than that in group 2 (8.3 ± 5.7 versus 2.7 ± 3.8, p < 0.001). Furthermore, fertilization, blastocyst, and pregnancy rates were significantly higher in group 1 than in group 2, respectively (61% versus 13.8%, p < 0.001; 32.8% versus 0%, p < 0.001; and 37.8% versus 0%, p < 0.001).

Conclusion: Strong evidence for overcoming primary impaired oocyte retrieval is still lacking in the literature. However, a 36-hour time interval between a second trigger and OPU seems ideal for FOPU cases. Therefore, communication between the physician and embryologist is crucial during OPU to detect such cases, especially in couples with a history of FOPU.

## Introduction

Ovum pick-up (OPU) is a procedure done during the treatment of infertility during which the ovum or oocytes are aspirated from the follicles in the ovaries of women. Failed oocyte pick-up (FOPU) is defined as the absence of oocytes retrieved from mature follicles after ovulation induction; the condition has also been referred to in the literature as empty follicle syndrome (EFS) [1]. Incidence ranges between 0.05% and 3.5% of in vitro fertilization (IVF) cycles [2,3]. Two types have been described, including genuine and false, and their occurrence is probably due to ovarian aging, yet the pathogenesis is unknown [4-7]. Several risk factors have been described, including high body mass index (BMI), low baseline luteinizing hormone (LH), hypogonadotropic hypogonadism, prolonged use of contraception, and duration of ovarian stimulation [8,9].

The time interval from ovulation induction to OPU was 32-36 hours [10,11]. Few studies reported the optimal trigger and OPU time interval. A study reported that the optimal trigger and OPU interval could be 36-37 hours in the antagonist protocol, 35-36 hours in the long protocol, and 35-37 hours in the flare-up protocol [12]. The present study objectives were to (1) compare secondary oocyte yield after FOPU at 24 vs. 36 hours post trigger and (2) assess associated fertilization, blastocyst, and pregnancy rates.

## Materials and methods

Study design, setting, participants, and sample size

**Figure 1:**
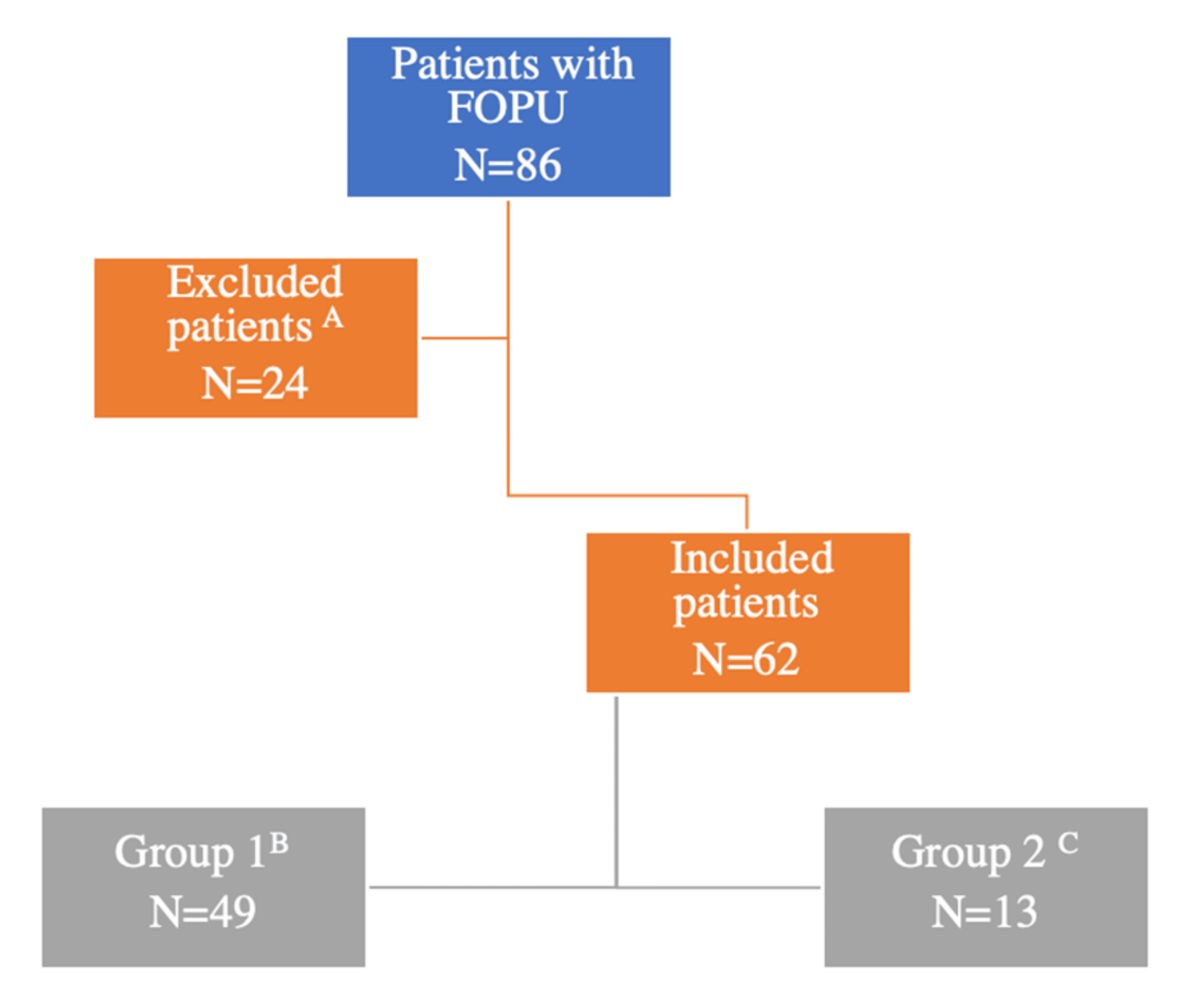
Flow chart explaining the number of included and excluded couples. ^A^Cycles with significant information unavailable in the patients’ medical files (number of follicles on trigger day, trigger medication, time interval between ovulation induction and oocyte pick-up, and number of retrieved oocytes), patient/medical error in trigger medication and timing, and low-response patients (≤5 follicles ≥ 14 mm the day of trigger). ^B^Patients who underwent a second OPU 36 hours after ovulation induction. ^C^Patients who underwent a second OPU 24 hours after ovulation induction. FOPU: failed oocyte pick-up; OPU: ovum pick-up.

A retrospective cohort single-center study was performed between July 2011 and April 2024, including 62 couples with second ovulation induction and OPU following FOPU in IVF cycles at Clinique Ovo, a private center affiliated with the University of Montreal. Group 1 comprises 49 patients who underwent a second OPU 36 hours after ovulation induction. Group 2, 13 patients, underwent a second OPU 24 hours after ovulation induction. Maternal age ranged between 20 and 43 years. The flow chart illustrates the number of included and excluded cases (Figure 1).

Inclusion criteria

The inclusion criteria include all medical records of diagnosed cases with failed oocyte pickup, including couples diagnosed with male factor infertility, low ovarian reserve, unexplained female infertility, polycystic ovarian syndrome (PCOS), and genetic disease. Fertility preservation cases were also included.

Exclusion criteria

The exclusion criteria include patients with incomplete medical record information including the absence of a description of the number of follicles on the trigger day, trigger medication, time interval between ovulation induction and oocyte pick-up, and number of retrieved oocytes. Patient or medical error in trigger medication administration or timing was also excluded. Low-response patients (≤5 follicles ≥ 14 mm the day of trigger) were not included.

Data collection and management

The variables extracted and analyzed were age, anti-Mullerian hormone (AMH), BMI, prior use of contraception, type of stimulation protocol, days of stimulation, primary and secondary ovulation induction medications used, estradiol and progesterone levels at trigger, number of follicles ≥ 14 mm, number of retrieved oocytes, number of mature oocytes, fertilization, cleavage, blastocyst, and pregnancy rates. Based on the time interval between the second ovulation induction and OPU, patients were categorized into two groups: a 36-hour group (group 1) and a 24-hour group (group 2).

Variables

A total of 16 variables were selected and analyzed (age, AMH, BMI, prior use of contraception, type of stimulation protocol, days of stimulation, type of trigger medication used, estradiol and progesterone levels at trigger, number of follicles ≥ 14 mm, number of retrieved oocytes, number of mature oocytes, fertilization, cleavage, blastocyst, and pregnancy rates).

Bias

Bias was minimized by using only complete data of patients and removal of low-response patients (≤5 follicles ≥ 14 mm the day of trigger) to ensure data reliability.

Failed and successful OPU procedures

Female partners mainly were stimulated using an antagonist protocol. Some cases were stimulated using a short or long agonist protocol (N = 18). Ovulation was triggered by administering human chorionic gonadotropin (hCG), gonadotropin-releasing hormone agonist (GnRH agonist), or both. At 36-hour post ovulation trigger administration, FOPU was diagnosed after failure to obtain oocytes in four or five follicular aspirations. Confirmation of diagnosis was achieved by having negative or low hCG (follicular or blood test) in cases triggered by hCG or double trigger (hCG + GnRH agonist). On the other hand, estradiol, LH, and progesterone levels were obtained in cases triggered by GnRH agonist. Second ovulation induction was performed by administering hCG or double trigger either 36 hours prior to the second OPU (on the night of the first OPU) or 24 hours prior to the second OPU (directly after the first OPU). At the time of the second OPU, the follicles are usually reconstituted, and their serohemorrhagic content appears gray on the ultrasound. The practice is to repuncture all the follicles. Oocytes were aspirated from the follicles in the ovaries by transvaginal ultrasound-guided puncture into the Global Total™ medium (Cooper-Origio, USA) in two groups (groups 1 and 2). Oocyte denudation was carried out using Cumulase™ (Cooper-Origio), and oocytes were assessed for maturity prior to intracytoplasmic sperm injection (ICSI). Sperm selection was performed using density gradient centrifugation or Zymot™, and fresh or frozen-warmed sperm was used. Only motile spermatozoa were immobilized before injection into mature oocytes. Embryo culture was performed in Global Total™, and embryo transfer was performed fresh on days 2, 3, and 5 or in a frozen embryo transfer cycle.

Data analysis

Statistical calculations were performed using SPSS version 29 (IBM Corp., Armonk, NY, USA). Following assessment of the normal distribution for continuous independent variables, means were expressed with standard deviations and compared by t-test or ANOVA or linear regression. Independent binary variables including maturation (MII/oocytes), fertilization (2PN/MII), blastocyst development (blastocyst/2PN), and pregnancy rate (hCG+/transfer) were compared between two time intervals (24 and 36 hours) by the chi-squared test or logistic regression. The null hypothesis was rejected with a p-value of less than 0.05.

## Results

Of the 62 couples with FOPU between July 2011 and April 2024, we compared 49 patients from group 1 and 13 from group 2. For group 1, the mean number of mature oocytes retrieved was significantly higher than that of group 2 (8.3 ± 5.7 vs. 2.7 ± 3.8, p < 0.001). Similarly, fertilization, blastocyst, and pregnancy rates were significantly higher in group 1 than group 2, respectively (61% vs. 13.8%, p < 0.001; 32.8% vs. 0%, p < 0.001; and 37.8% vs. 0%, p < 0.001). However, the use of agonist protocol, BMI, and the prior use of contraception were not statistically significant. The use of GnRH agonist alone as primary ovulation induction was not significantly higher in group 1 compared to group 2 (26.5% vs. 23.0%, p = 0.79). It was found that 42.3% (n = 22/62) of patients triggered with hCG 5,000 IU had a BMI > 35. The types of second trigger and all the results are summarized in Tables 1, 2, respectively.

**Table 1:** Types of second trigger. Group 1: 36 hours prior to the 2nd OPU (on the night of 1st OPU); group 2: 24 hours prior to the 2nd OPU (directly after the 1st OPU); hCG: human chorionic gonadotropin; IU: international unit. Suprefact® is the brand name for buserelin, a GnRH agonist. Ovidrel® is the brand name for choriogonadotropin alfa, a recombinant form of hCG.

	Group 1: 36 hours (N = 49)	Group 2: 24 hours (N = 13)
hCG 10,000 IU	32/49 (65.4%)	8/13 (61.6%)
hCG 5,000 IU	10/49 (20.4%)	3/13 (23%)
hCG 15,000 IU	0/49 (0%)	1/13 (7.7%)
Ovidrel® 250 IU	2/49 (4%)	0/13 (0%)
Ovidrel® 250 IU + Suprefact	0/49 (0%)	1/13 (7.7%)
Suprefact® + hCG 10,000 IU	4/49 (8.2%)	0/13 (0%)
Suprefact® + hCG 5,000 IU	1/49 (2%)	0/13 (0%)

**Table 2:** Results of group 1 and group 2. *Statistically significant with a p-value of <0.05. Group 1: patients with a second trigger 36 hours prior to the 2nd OPU; group 2: patients with a second trigger 24 hours prior to the 2nd OPU; AMH: anti-Mullerian hormone; BMI: body mass index; fertilization rate: number of 2 pronuclei divided by the number of mature oocytes; cleavage rate: number of cleaved embryos divided by the number of 2 pronuclei; blastocyst rate: number of blastocysts divided by the number of 2 pronuclei; pregnancy rate: number of pregnancies divided by the number of transfers. The chi-squared test and t-test were performed for the variables.

	Group 1: 36 hours (N = 49)	Group 2: 24 hours (N = 13)	p-value	Statistical test/value
Age	34.6 ± 4.9	34.9 ± 6.9	0.88	t-test/-0.19
AMH (ng/mL)	3.8 ± 3.1	2.1 ± 1.6	0.004*	t-test/2.47
Days of stimulation	11.9 ± 1.9	12.8 ± 1.8	0.11	t-test/-1.83
Estradiol at trigger (pmol/L)	12,092 ± 5,428	12,750 ± 7,078	0.75	t-test/0.40
Progesterone at trigger (nmol/L)	3.75 ± 1.8	3.44 ± 1.0	0.41	t-test/0.76
Number of follicles (≥14 mm)	14.3 ± 7.4	15.8 ± 7.2	0.52	t-test/-0.77
Number of oocytes retrieved	10.4 ± 7.1	5.4 ± 4.0	0.001*	t-test/3.12
Number of mature oocytes	8.3 ± 5.7	2.7 ± 3.8	<0.001*	t-test/4.22
Fertilization rate	61%	13.8%	<0.001*	Chi-squared/3.61
Cleavage rate	96.5%	100%	0.64	Chi-squared/-0.99
Blastocyst rate	32.8%	0%	<0.001*	Chi-squared/3.06
Pregnancy rate	37.8%	0%	<0.001*	Chi-squared/3.43
Agonist protocol (long/short)	14/49 (28.5%)	4/13 (30.7%)	0.87	Chi-squared/-0.20
Prior use of contraception	6/49 (12.2%)	2/13 (15.3%)	0.76	Chi-squared/-0.33
BMI > 35	23/49 (46.9%)	7/13 (53.8%)	0.65	Chi-squared/-0.53
BMI ≤ 22.9	12/44 (27.2%)	5/11 (45.4%)	0.08	Chi-squared/-1.44

## Discussion

This is the first study to explore the effect of the ovulation trigger time interval after FOPU. The 36-hour time interval was found to be associated with a greater number of oocytes retrieved, as well as greater maturation, fertilization, blastocyst formation, and pregnancy rates. Shen et al. [13] in a retrospective study involving 4,673 patients found that the time interval from trigger to oocyte collection in order to obtain over 80% of oocyte maturity varied according to the ovarian stimulation protocol: GnRH antagonist protocol (34.5-36.3 hours), the short protocol (36.0-37.7 hours), and the long agonist protocol (35.0-39.7 hours) [13]. Rescue trigger using 10,000 IU hCG was introduced by Ndukwe et al. [14] in 1997. This hCG dosage can be used in both agonist and antagonist protocols [15,16]. Our study showed successful oocyte retrieval following various modalities of the second trigger (Table 1). Nonetheless, more than half of the cases were triggered by 10,000 IU hCG.

Among the various predisposing factors for FOPU, a previous history of FOPU has been reported to be associated with a 15%-20% risk of recurrence [17]. Inadequate timing or dose of trigger is also associated with the risk of FOPU. We have excluded seven cases of FOPU in our study due to patient or medical error in trigger medication and timing to optimize the analysis of our data and decrease the risk of bias. Similarly, the elimination of low-response patients (six cases of FOPU) was important as they would have a higher probability of FOPU leading to an increased risk of bias.

Other risk factors include high or low BMI, low baseline LH, hypogonadotropic hypogonadism, and prolonged use of contraception prior to GnRH agonist trigger alone [8,9]. We could not evaluate the effect of baseline LH as it was not available for most of the included patients, which is a limitation of our study. The prior use of contraception could not be adequately evaluated in our study. BMI < 22 has been reported to increase the chances of FOPU when using an agonist trigger [8]. Another study did not find a correlation [18]. Our findings agree with the latter study as we did not find a statistical significance in patients with low BMI. On the other hand, we found that 42.3% of patients with FOPU who were triggered with hCG 5,000 IU had BMI > 35, which may be a factor due to inadequate absorption or dosage of the medication.

Aspiration pressure for oocyte retrieval has been reported to be a factor for FOPU [19,20], but in our center, we use an aspiration pressure not higher than 150 mmHg due to the increased possibility of oocyte damage above that level. Communication between the physician, embryologist, nurse, and patient is crucial in case of suspicion of FOPU. The ideal pathway is to stop follicular fluid aspiration when three to five mature follicles (>14 mm) have been aspirated and no oocytes have been retrieved. Discussion and explanation with the patient are essential as it is stressful for couples at this stage. Performing follicular fluid pregnancy tests and serum hCG could be helpful in cases triggered by hCG. Furthermore, LH levels < 15 mIU/mL and progesterone < 3.5 ng/mL eight to 12 hours post trigger could help prevent FOPU when GnRH agonists are used as a trigger [21]. In our study, all of our cases triggered with hCG had a negative or low hCG (data not shown). The LH test for patients triggered with GnRH agonist on the day of OPU was <15 mIU/mL whenever available in the patients’ files.

To our knowledge, this is the first study evaluating the time interval between a second ovulation induction and OPU in FOPU. The strengths of our study include a larger sample size compared to other reports published in the literature, strict inclusion criteria including the elimination of medical/patient error of trigger dose or timing of administration, cycles with significant absence of information, and low-response patients.

Limitations

The limitations of our study include the difficulty in concluding contraception use as a factor due to the undocumented duration of use, the high number of eliminated cases (N = 24), sample size, and unequal distribution of the two groups.

## Conclusions

Strong evidence for overcoming primary impaired oocyte retrieval is still lacking in the literature. However, a 36-hour time interval between a second trigger and OPU seems advantageous for FOPU cases. Therefore, communication between the physician and embryologist is crucial during OPU for the early detection of such situations, especially in couples with a history of FOPU.
